# *Fusobacterium nucleatum* Abundance is Associated with Cachexia in Colorectal Cancer Patients: The ColoCare Study

**DOI:** 10.1002/cam4.70431

**Published:** 2024-11-25

**Authors:** Mmadili N. Ilozumba, Tengda Lin, Sheetal Hardikar, Doratha A. Byrd, June L. Round, W. Zac Stephens, Andreana N. Holowatyj, Christy A. Warby, Victoria Damerell, Christopher I. Li, Jane C. Figueiredo, Adetunji T. Toriola, David Shibata, Gary C. Fillmore, Bartley Pickron, Erin M. Siegel, Christoph Kahlert, Vaia Florou, Biljana Gigic, Jennifer Ose, Cornelia M. Ulrich

**Affiliations:** ^1^ Huntsman Cancer Institute Salt Lake City Utah USA; ^2^ Department of Population Sciences University of Utah USA; ^3^ H. Lee Moffitt Cancer Center and Research Institute Tampa Florida USA; ^4^ Division of Microbiology and Immunology, Department of Pathology University of Utah School of Medicine Salt Lake City Utah USA; ^5^ Vanderbilt University Medical Center Nashville Tennessee USA; ^6^ Department of General, Visceral, and Transplantation Surgery Heidelberg University Hospital Heidelberg Germany; ^7^ Fred Hutchinson Cancer Center Seattle WA USA; ^8^ Department of Medicine, Cedars‐Sinai Medical Center Samuel Oschin Comprehensive Cancer Institute Los Angeles California USA; ^9^ Washington University School of Medicine in St. Louis St. Louis Missouri USA; ^10^ Department of Surgery University of Tennessee Health Science Center Memphis Tennessee USA; ^11^ Department of Information and Communication, Faculty for Media, Information and Design University of Applied Sciences and Arts Hannover Germany

**Keywords:** cachexia, colorectal cancer, *Fusobacterium nucleatum*

## Abstract

**Background:**

Cachexia accounts for about 20% of all cancer‐related deaths and indicates poor prognosis. The impact of *Fusobacterium nucleatum (Fn*), a microbial risk factor for colorectal cancer (CRC), on the development of cachexia in CRC has not been established.

**Methods:**

We evaluated the association between *Fn* abundance in pre‐surgical stool samples and onset of cachexia at 6 months post‐surgery in *n* = 87 patients with stages I–III CRC in the ColoCare Study.

**Results:**

High fecal *Fn* abundance compared to negative/low fecal *Fn* abundance was associated with 4‐fold increased risk of cachexia onset at 6 months post‐surgery (OR = 4.82, 95% CI = 1.15, 20.10, *p* = 0.03).

**Conclusion:**

Our findings suggest that high fecal *Fn* abundance was associated with an increased risk of cachexia at 6 months post‐surgery in CRC patients. This is the first study to link *Fn* abundance with cachexia in CRC patients, offering novel insights into biological mechanisms and potential management of cancer cachexia. Due to the small sample size, our results should be interpreted with caution. Future studies with larger sample sizes are needed to validate these findings.

Cancer cachexia is a multifaceted metabolic syndrome marked by a persistent decline in skeletal muscle mass due to cancer and systemic inflammation, distinct from age‐related sarcopenia, which may also involve the loss of fat mass, while being resistant to nutritional or physical interventions [1, 2]. Cachexia contributes to nearly 20% of all cancer‐related deaths and is reflective of poor prognosis, functional decline, and heightened risk of toxicity from chemotherapy [1, 3, 4, 5, 6]. Cachexia affects ~50% of patients with colorectal cancer (CRC) [7], the third leading cause of cancer‐related deaths in the US [8]. Our current understanding of the underlying mechanisms of cachexia onset is still limited. However, recent hypotheses suggest that the gut microbiome, a vast community of microorganisms, may play a significant role in the development of cancer cachexia by inducing systemic inflammation [9, 10].

*Fusobacterium nucleatum* (*Fn*), a gram negative, non‐spore forming anaerobic bacterium, commensal to the human oral cavity, has emerged as a key microbial player in accelerating CRC development [11, 12, 13, 14, 15]. Both fecal *Fn* abundance and *Fn* abundance in the tumor may influence the tumor microenvironment and have been implicated in CRC recurrence, poorer prognosis, and chemotherapy resistance [16, 17, 18, 19, 20, 21]. However, to the best of our knowledge, no study has examined the association between *Fn* abundance and cachexia onset in CRC patients despite substantial biological plausibility (Figure 1).

**FIGURE 1:**
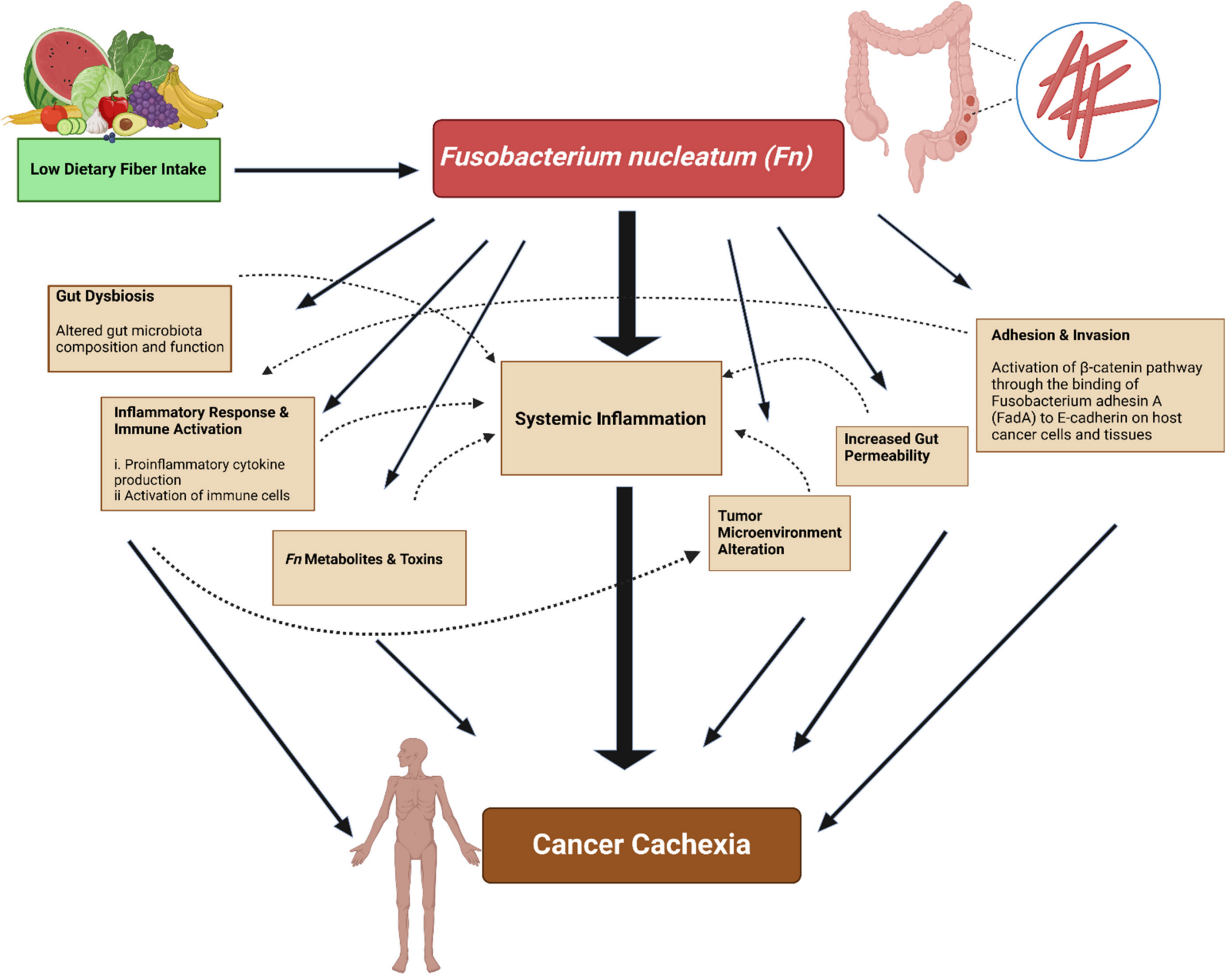
Putative mechanisms linking *Fusobacterium nucleatum* (Fn) to cancer cachexia.

Therefore, the objective of this study is to investigate the association of *Fn* abundance in stool samples collected prior to surgery with onset of cachexia at 6 months post‐surgery within a prospective cohort of CRC patients.

Study participants are from the prospective ColoCare Study (ClinicalTrials.gov NCT02328677) [22]. The present study comprises data collected from *n* = 87 patients (39 cachectic and 48 non‐cachectic, ICD‐10‐CM R64) diagnosed with stage I‐III CRC (ICD‐10‐CM C18, C18.0, C18.2‐C20) at two study sites at the Heidelberg University Hospital ([HD], Germany, *n* = 58) and the Huntsman Cancer Institute ([HCI], Utah, USA, *n* = 29) with pre‐surgery available stool samples. Our study focused on stage I‐III CRC to examine cachexia in early‐stage CRC patients. The study was approved by the institutional review boards of the respective institutions, and all patients provided written informed consent. A detailed description of the study population is provided in Supporting Information S1 (available online).

Relative abundance of *Fn* was calculated in proportion to total bacterial load as the ratio of (2^ [− *Fn*]/ 2^ [− 16S]) × average *Ct* values [23, 24]. As described in prior studies investigating *Fn* abundance, patients with positive (detectable) *Fn* DNA fecal biospecimen was classified into *Fn* high and *Fn* low groups based on median *Fn Ct* values for each study site (HD: low < 0.034; high > 0.034; HCI: low < 0.00067; high > 0.00067) to account for geographic differences and influence of geography on the fecal microbiome [20, 23, 25, 26, 27, 28]. Patients with *Fn* negative (undetectable) abundance were included in the *Fn* low group [23, 29]. A detailed description of the experimental methods is provided in Supporting Information S1 (available online).

Cachexia was assessed at 6 months post‐surgery. Using Fearon criteria, cachexia was defined as > 5% weight loss over the past 6 months or body mass index (BMI) of < 20 kg/m^2^ and weight loss of > 2% [1]. Multivariable logistic regression was used to estimate the associations between *Fn* abundance with onset of cachexia while adjusting for important covariates including age based on median age at diagnosis (< 65 years old and ≥ 65 years old), stage at diagnosis (I and II, III), tumor site (colon, rectum), and recruitment center (HD, Germany, or HCI, USA) based on stepwise selection procedure. A detailed description of the stepwise selection procedure is provided in Supporting Information S1 (available online). Sensitivity analyses were conducted additionally adjusting for antibiotic use in the past year; excluding patients with antibiotics use in the past year (*n* = 17) and patients who received neo‐adjuvant treatment (*n* = 23), respectively; stratifying analysis by recruitment site (HCI, HD); and examining the associations between *Fn* abundance with onset of cachexia, using “*Fn* negative” as the reference level. Statistical significance was defined as *p* < 0.05 and all statistical tests were 2‐sided. We also considered 0.05 ≤ *p* < 0.10 as suggestive statistical significance. All statistical analyses were conducted using SAS version 9.4 statistical software (SAS Institute, Inc).

Table 1 presents the demographic, clinical, and pathological characteristics of the study participants. Study participants included 39 (45%) cachectic and 48 (55%) non‐cachectic CRC patients at 6 months post‐surgery. Among cachectic patients, 28 (72%) had rectal cancer and 11 (28%) had colon cancer, whereas among non‐cachectic patients, 18 (38%) had rectal cancer, whereas 30 (63%) had colon cancer (*p* < 0.001).

**TABLE 1:** Study population characteristics, *n* = 87.

Characteristics	Cachectic at 6 months post‐surgery, *n* (%)	Non‐cachectic at 6 months post‐surgery, *n* (%)	*p*
	39 (45)	48 (55)	
Age at diagnosis, *n* (%)^a^			0.22
< 65	21 (54)	32 (67)	
≥ 65	18 (46)	16 (33)	
Patient sex, *n* (%)^a^			0.55
Female	17 (44)	24 (50)	
Male	22 (56)	24 (50)	
Race, *n* (%)^b^			0.24
White	39 (100)	44 (92)	
Asian	0 (0)	1 (2)	
Other	0 (0)	3 (6)	
Ethnicity, *n* (%)^b^			1.00
Non‐Hispanic	39 (100)	47 (98)	
Hispanic	0 (100)	1 (2)	
Study site, *n* (%)^a^			0.01
HCI	7 (18)	22 (46)	
HD	32 (82)	26 (54)	
*Fusobacterium nucleatum* (*Fn*) abundance, *n* (%)^a^, ^c^			0.12
*Fn*‐Negative/Low	29 (74)	42 (88)	
*Fn*‐High	10 (26)	6 (13)	
Stage at diagnosis, *n* (%)^a^			0.29
I	11 (28)	7 (15)	
II	11 (28)	17 (35)	
III	17 (44)	24 (50)	
Tumor site, *n* (%)^a^			0.001
Colon	11 (28)	30 (63)	
Rectum	28 (72)	18 (38)	
Smoking status, *n* (%)^b^			0.10
Non‐smoker	18 (46)	20 (42)	
Former smoker	18 (46)	16 (33)	
Current smoker	3 (8)	12 (25)	
BMI at baseline, kg/m^2^, *n* (%)^b^			0.046
< 25	12 (31)	16 (34)	
25‐ < 30	23 (59)	17 (36)	
≥ 30	4 (10)	14 (30)	
Physical activity, MET h/week, *n* (%)^a^			0.49
Active, ≥ 8.75	15 (38)	22 (46)	
Inactive, < 8.75	24 (62)	26 (54)	
CRP, mg/L, *n* (%)^b^			0.099
Low level, < 10 mg/L	35 (90)	36 (75)	
High level, > = 10 mg/L	4 (10)	12 (25)	
Neo‐adjuvant treatment, *n* (%)^a^			0.07
No	25 (64)	39 (81)	
Yes	14 (36)	9 (19)	
Adjuvant treatment, *n* (%)^a^			0.24
No	26 (67)	26 (54)	
Yes	13 (33)	22 (46)	
NSAID/Aspirin use, *n* (%)^b^			0.76
No	33 (89)	37 (86)	
Yes	4 (11)	6 (14)	
Antibiotic use, *n* (%)^b^			0.02
No	7 (86)	9 (36)	
Yes	1 (13)	16 (64)	
Dietary fiber intake, grams/day, *n* (%)^a^			0.99
Low fiber	25 (74)	28 (74)	
High fiber	9 (26)	10 (26)	

*Note:* Missing data: aspirin use: *n* = 7; BMI, *n* = 1; dietary fiber intake, *n* = 15; antibiotic use, *n* = 54.

Abbreviations: CRP, C‐reactive protein (CRP), mg/L; HCI, Huntsman Cancer Institute; HD, Heidelberg; MET, metabolic equivalent per tasks.

^a^ Chi square test.

^b^ Fisher's exact test.

^c^ *Fn* abundance was defined as the presence of detectable *Fn* DNA abundance (*Fn* positive) in fecal biospecimens. Patients were classified into *Fn* high and *Fn* low groups based on the median *Fn* Ct values specific to each study site (HD: low < 0.034, high > 0.034; HCI: low < 0.00067, high > 0.00067). Patients with undetectable *Fn* abundance (*Fn* negative) were included in the *Fn* low group.

Association of fecal *Fn* abundance with onset of cachexia at 6 months post‐surgery in CRC patients is presented in Table 2. High fecal *Fn* abundance compared to negative/low fecal *Fn* abundance was associated with iia 4‐fold increased risk of cachexia onset at 6 months post‐surgery (OR = 4.82, 95% CI = 1.15, 20.10, *p* = 0.03). Sensitivity analyses additionally adjusting for antibiotic use in the past year (Table S1); excluding patients with antibiotic use in the past year (Table S2) or patients who received neo‐adjuvant treatment (Table S3) did not change the study findings. In the stratified analysis by recruitment site, suggestive stronger positive association was observed in HD (OR = 7.00, 95% CI = 0.80, 61.15; *p* = 0.06) (Table S4). High fecal *Fn* abundance compared to negative fecal *Fn* abundance was associated with a suggestive 3‐fold increased risk of cachexia onset at 6 months post‐surgery (OR = 3.62, 95% CI = 0.82, 15.95, *p* = 0.09), whereas low fecal *Fn* abundance compared to negative fecal *Fn* abundance was associated with a suggestive 71% decreased risk of cachexia onset at 6 months post‐surgery (OR = 0.29, 95% CI = 0.07, 1.10, *p* = 0.07) (Table S5).

**TABLE 2:** Association between *Fusobacterium nucleatum* (*Fn*) abundance^a^ and onset of cachexia at 6 months post‐surgery in colorectal cancer patients, *n* = 87 (39 [45%] cachectic/48 [55%] non‐cachectic patients).

	*n* (%)	OR (95% CI)^b^	*p*
*Fn*‐negative/low	71 (82)	Ref	
*Fn*‐high	16 (18)	4.82 (1.15, 20.10)	**0.03**

*Note:* Bold values indicate *p*‐values less than 0.05, reflecting statistical significance.

^a^ *Fn* abundance was defined as the presence of detectable *Fn* DNA abundance (*Fn* positive) in fecal biospecimens. Patients were classified into *Fn* high and *Fn* low groups based on the median *Fn* Ct values specific to each study site (HD: low < 0.034, high > 0.034; HCI: low < 0.00067, high > 0.00067). Patients with undetectable *Fn* abundance (*Fn* negative) were included in the *Fn* low group.

^b^ Adjusted for age based on median age at diagnosis (< 65 years old, ≥ 65 years old), stage at diagnosis (I and II, III), tumor site (colon, rectum), recruitment center (Heidelberg University Hospital, Germany or Huntsman Cancer Institute, Salt Lake City).

Epidemiological studies examining the association between *Fn* abundance and cancer cachexia in CRC patients are currently lacking. To our knowledge, this is the first investigation of *Fn* abundance in the context of cachexia onset. Our findings indicate that patients with pre‐surgical high fecal *Fn* abundance have an increased risk of developing cachexia within six months post‐surgery compared to those with negative or low fecal *Fn* abundance. This supports the study hypothesis that high abundance of *Fn* plays a mechanistic role in the onset of cachexia in CRC patients. *Fn* is a gram negative, non‐spore forming anaerobic bacterium, ubiquitous in the human oral cavity and has been delineated as an oncogenic bacterium [11]. Several studies have shown that fecal *Fn* abundance or *Fn* abundance in the tumor play a significant role in promoting CRC risk [11, 12, 13, 14, 15], as well as CRC recurrence, poorer prognosis, and chemotherapy resistance [11, 16, 17, 18, 19, 20, 21]. *Fn* abundance may influence the tumor microenvironment as tumors with high *Fn* abundance have been shown to have lower T cell density, thus dysregulating anti‐tumor immunity [11, 16]. A characteristic feature of *Fn* is the presence of adhesins, which are virulence factors enabling the bacteria to invade and attach to host cells, thereby promoting tumorigenesis [30]. Specifically, the activation of β‐catenin pathway through the binding of Fusobacterium adhesin A (FadA) to E‐cadherin on host cells has been hypothesized [31]. In an ApcMin/+ model, *Fn*‐fed mice compared to sham‐fed mice exhibited intratumoral myeloid cells, including macrophages, dendritic cells and myeloid‐derived suppressor cells as well as the activation of the NF‐κB pathway and stimulated the expression of the genes encoding several pro‐inflammatory cytokines, including TNF, IL‐6, IL‐8, and IL‐1β [32].

Furthermore, *Fn* metabolites have been shown to elicit a pro‐inflammatory milieu by the upregulation of toll‐like receptor 4 (TLR4) and Myc and genes encoding pro‐inflammatory cytokines in Caco‐2 cells [33]. Similarly, proinflammatory cytokines have been implicated in the onset of cachexia, a severe condition characterized by systemic inflammation [34, 35]. Although the present study defined cancer cachexia using Fearon's criteria, cancer cachexia has also been defined using a diagnostic criterion that involves systemic inflammation, which is detected by abnormal biochemical markers [36, 37, 38, 39, 40, 41]. Hence, underscoring the potential effect of *Fn* in inducing systemic inflammation and cancer cachexia. *Fn* synthesizes butyrate, a short‐chain fatty acid (SCFA) through the fermentation of non‐digestible dietary fibers [42], which may influence gut health [43, 44]. Impaired gut barrier integrity can lead to increased gut permeability, with resultant leakage of harmful substances such as toxins and bacteria in the bloodstream, triggering immune responses and inflammation. Bacterial toxins, for example, lipopolysaccharide (LPS) in circulation can induce systemic inflammation and metabolic changes, potentially exacerbating muscle wasting and weight loss associated with cachexia [45, 46]. Butyrate acts as an anti‐inflammatory metabolite and plays a key role in intestinal homeostasis and cellular energy metabolism as well as inhibits the progression of CRC cells by regulating the inhibition of histone deacetylases (HDAC) and NF‐κB proinflammatory signaling pathways [42, 47, 48]. However, there is a conundrum with existing evidence showing that the oncogenic *Fn* actively synthesizes butyrate [49]. The bioactivity of butyrate in CRC tumors is not fully understood, raising questions about why *Fn* known for its oncogenic properties, can synthesize butyrate, an anti‐inflammatory metabolite [50, 51]. There is evidence that *Fn* may require a disruption in the gut microbiome to induce and sustain inflammatory processes [52], by impacting the growth of neighboring species [53, 54]. Whether *Fn* and *Fn* metabolites interact with proinflammatory cytokines to influence cachexia onset in CRC patients should be explored in future studies. Additionally, further investigation into the impact of *Fn* on the composition and structure of the gut microbiome and their combined effects on cachexia onset is warranted.

We observed a higher incidence of cachexia in rectal cancer patients compared to those with colon cancer, though the underlying reason is unclear. We hypothesize that differences in tumor biology, location, and treatment approaches may influence systemic inflammation and metabolic changes. Rectal cancer patients often undergo more aggressive treatments, such as neoadjuvant therapy, which could contribute to the increased incidence of cachexia. In contrast, a Japanese retrospective cohort study of patients with advanced CRC and metastasis reported higher cachexia incidence in colon vs. rectal cancer patients after six months of systemic chemotherapy [55]. Geographic location, stage at diagnosis, and treatment differences may explain the discrepancies between studies. Further research is needed to better understand cachexia incidence by CRC tumor site. In our stratified analysis by recruitment site, we observed a suggestively stronger positive association among German participants. The differences between German and US participants may be due to lifestyle, dietary, and cultural differences across these geographic regions, which can influence the microbiome.

Our study had several strengths. To date, this is the first study leveraging fecal samples collected prior to surgery and without any antibiotic use or other interventions to investigate the associations of *Fn* abundance with cachexia onset six months post‐surgery in CRC patients. One of the major strengths of our study is its prospective and longitudinal design, which facilitated the use of meticulous and high‐standard collection procedures for at‐home collected samples prior to surgery and allowed for a comprehensive six‐month follow‐up period to monitor the onset of cachexia in patients. Fecal sampling is non‐invasive and, since *Fn* is present in fecal samples, it may serve as a valuable biomarker for the prediction of cachexia onset in CRC patients. We calculated the relative abundance of *Fn* as the ratio of *Fn* to total 16S rRNA as demonstrated in previous studies [23, 24], which showed that considering the ratio of *Fn* to other bacterial strains in feces‐based qPCR assays provides better prognostic value than measuring *Fn* alone. The abundance of *Fn* was quantified using advanced standardized protocols for biospecimen collection, processing, storage, and analyses.

The study has a few limitations. Due to the small sample size, we observed large odds ratios and wide confidence intervals. Our findings require validation in future studies with larger sample sizes. Antibiotic use was assessed by a different instrument that was not completed by all participants; hence, we had information on antibiotic use for a subset of the participants. Antibiotic use influences the gut microbiome by reducing microbial diversity and inducing changes in microbial function [56]. However, adjusting for antibiotic use in the fully adjusted logistic model or excluding the few participants with information on antibiotic use in sensitivity analyses did not change result estimates. The generalizability of the results to other racial and ethnic groups is limited, as the study participants were predominantly White patients from the US and German sites, despite covering different geographical regions. Assessing cachexia immediately after surgery can be challenging due to potential post‐operative inflammatory responses, which might influence cachexia markers. Nevertheless, we believe the use of Fearon's criteria was appropriate, as it is widely accepted for identifying cancer cachexia. Further research is needed to explore this area in greater detail. Although cachexia is more commonly associated with advanced‐stage cancer, our study focused on stages I‐III to examine cachexia in early‐stage CRC. Further studies including stage IV patients would be valuable for understanding the broader spectrum of cachexia development.

In conclusion, we observed that pre‐operative high fecal *Fn* abundance was associated with an increased risk of cachexia at six months post‐surgery in CRC patients. Future studies with larger sample sizes are needed to validate our findings and further examine the biological mechanisms underlying cachexia onset. Our findings suggest that *Fn* holds potential as a prognostic indicator for cachexia and as a promising target for developing strategies aimed at its prevention and treatment. Further research into its role could lead to novel insights and therapeutic approaches in the management of cachexia in clinical settings.

## Author Contributions

**Mmadili N. Ilozumba:** conceptualization, methodology, software, project administration, formal analysis, visualization, writing – review and editing, writing – original draft, investigation. **Tengda Lin:** methodology, software, project administration. **Sheetal Hardikar:** project administration, investigation, writing – review and editing. **Doratha A. Byrd:** investigation, writing – review and editing, funding acquisition. **June L. Round:** data curation, investigation, writing – review and editing, methodology, resources. **W. Zac Stephens:** methodology, investigation, data curation, resources, writing – review and editing. **Andreana N. Holowatyj:** funding acquisition, writing – review and editing, methodology, resources. **Christy A. Warby:** data curation, investigation, writing – review and editing. **Victoria Damerell:** project administration, writing – review and editing, investigation. **Christopher I. Li:** funding acquisition, writing – review and editing, investigation. **Jane C. Figueiredo:** writing – review and editing, investigation, funding acquisition. **Adetunji T. Toriola:** investigation, funding acquisition, writing – review and editing. **David Shibata:** investigation, funding acquisition, writing – review and editing. **Gary C. Fillmore:** investigation, writing – review and editing. **Bartley Pickron:** conceptualization, writing – review and editing. **Erin M. Siegel:** funding acquisition, writing – review and editing, investigation. **Christoph Kahlert:** investigation, writing – review and editing. **Vaia Florou:** investigation, writing – review and editing. **Biljana Gigic:** investigation, funding acquisition, project administration, writing – review and editing, resources, data curation. **Jennifer Ose:** conceptualization, investigation, supervision, writing – review and editing. **Cornelia M. Ulrich:** conceptualization, data curation, funding acquisition, investigation, writing – review and editing, resources, supervision.

## Conflicts of Interest

C.M.U. has as cancer center director oversight over research funded by several pharmaceutical companies, but has not received funding directly herself. The remaining authors declare no conflict of interest.

## Supporting information


Table S1.

Supporting Information S1.

## Data Availability

ColoCare Study data is available from the corresponding author on reasonable request and as described on the ColoCare website (https://uofuhealth.utah.edu/huntsman/labs/colocare‐consortium/).
