# Supplementary material for: Fusobacterium nucleatum Abundance is Associated with Cachexia in Colorectal Cancer Patients: The ColoCare Study
Source: Cancer Med. 2024 Nov 25;13(22):e70431. doi: 10.1002/cam4.70431 (PMC11588854; doi:10.1002/cam4.70431)
Supplement: Supplementary file 1 — Table S1. [file CAM4-13-e70431-s002.docx]

**Supplemental Table 1**: Association between *Fusobacterium nucleatum* (*Fn*) abundance^a^ and onset of cachexia at 6 months post-surgery in colorectal cancer patients additionally adjusting for antibiotic use in the past year, n= 87 (39 [45%] cachectic/48 [55%] non-cachectic patients)

|  | n (%) | OR (95% CI)^b^ | p-value |
| --- | --- | --- | --- |
| *Fn*-Negative/Low | 71 (82) | Ref |  |
| *Fn*-High | 16 (18) | 5.19 (1.02, 26.35) | **0.047** |

^a^*Fn* abundance was defined as the presence of detectable *Fn* DNA abundance (*Fn* positive) in fecal biospecimens. Patients were classified into *Fn* high and *Fn* low groups based on the median *Fn* Ct values specific to each study site (HD: low < 0.034, high > 0.034; HCI: low < 0.00067, high > 0.00067). Patients with undetectable *Fn* abundance (*Fn* negative) were included in the *Fn* low group.

^b^Adjusted for age based on median age at diagnosis (<65, ≥65), stage at diagnosis (I-II, III), tumor site (colon, rectum), recruitment center (Heidelberg University Hospital or Huntsman Cancer Institute, Salt Lake City), antibiotic use in the past year (no, yes)

**Supplemental Table 2**: Association between *Fusobacterium nucleatum* (*Fn*) abundance^a^ and onset of cachexia at 6 months post-surgery in colorectal cancer patients excluding patients with antibiotic use in the past year, n= 70 (38 [54%] cachectic/32 [46%] non-cachectic patients)

|  | n (%) | OR (95% CI)^b^ | p-value |
| --- | --- | --- | --- |
| *Fn*-Negative/Low | 58 (83) | Ref |  |
| *Fn*-High | 12 (17) | 6.78 (1.10, 41.75) | **0.04** |

^a^*Fn* abundance was defined as the presence of detectable *Fn* DNA abundance (*Fn* positive) in fecal biospecimens. Patients were classified into *Fn* high and *Fn* low groups based on the median *Fn* Ct values specific to each study site (HD: low < 0.034, high > 0.034; HCI: low < 0.00067, high > 0.00067). Patients with undetectable *Fn* abundance (*Fn* negative) were included in the *Fn* low group.

^b^Adjusted for age based on median age at diagnosis (<65, ≥65), stage at diagnosis (I-II, III), tumor site (colon, rectum), recruitment center (Heidelberg University Hospital or Huntsman Cancer Institute, Salt Lake City)

**Supplemental Table 3**: Association between *Fusobacterium nucleatum* (*Fn*) abundance^a^ and onset of cachexia at 6 months post-surgery in colorectal cancer patients excluding patients who received neo-adjuvant treatment, n= 64 (25 [39%] cachectic/39 [61%] non-cachectic patients)

|  | n (%) | OR (95% CI)^b^ | p-value |
| --- | --- | --- | --- |
| *Fn*-Negative/Low | 50 (78) | Ref |  |
| *Fn*-High | 14 (22) | 12.22 (1.29, 115.63) | **0.03** |

*^a^Fn* abundance was defined as the presence of detectable *Fn* DNA abundance (*Fn* positive) in fecal biospecimens. Patients were classified into *Fn* high and *Fn* low groups based on the median *Fn* Ct values specific to each study site (HD: low < 0.034, high > 0.034; HCI: low < 0.00067, high > 0.00067). Patients with undetectable *Fn* abundance (*Fn* negative) were included in the *Fn* low group.

^b^Adjusted for age based on median age at diagnosis (<65, ≥65), stage at diagnosis (I-II, III), tumor site (colon, rectum), recruitment center (Heidelberg University Hospital or Huntsman Cancer Institute, Salt Lake City)

**Supplemental Table 4.** Associations between *Fusobacterium nucleatum* (Fn) abundance^a^ and onset of cachexia at 6-months post-surgery stratified by recruitment site, n=87

|  |  | n (%) | no. of cases cachectic/non-cachectic |  | n (%) | OR (95% CI)^b^ | p-value |
| --- | --- | --- | --- | --- | --- | --- | --- |
| Study site |  |  |  |  |  |  |  |
|  | HCI | 29 (33) | 7/22 | *Fn*-Negative/Low | 21 (72) | Ref |  |
|  |  |  |  | *Fn*-High | 8 (28) | 2.55 (0.42, 15.41) | 0.36 |
|  | HD | 58 (67) | 32/26 | *Fn*-Negative/Low | 50 (86) | Ref |  |
|  |  |  |  | *Fn*-High | 8 (14) | 7.00 (0.80, 61.15) | **0.06** |

*^a^Fn* abundance was defined as the presence of detectable *Fn* DNA abundance (*Fn* positive) in fecal biospecimens. Patients were classified into *Fn* high and *Fn* low groups based on the median *Fn* Ct values specific to each study site (HD: low < 0.034, high > 0.034; HCI: low < 0.00067, high > 0.00067). Patients with undetectable *Fn* abundance (*Fn* negative) were included in the *Fn* low group.

^b^Fisher’s exact test due to sparse cells (frequency <5)

HCI: Huntsman Cancer Institute, HD: Heidelberg University Hospital

**Supplemental Table 5**: Association between *Fusobacterium nucleatum* (*Fn*) abundance*^a^* and onset of cachexia at 6 months post-surgery in colorectal cancer patients, n= 87 (39 [45%] cachectic/48 [55%] non-cachectic patients)

|  | n (%) | OR (95% CI)^b^ | p-value |
| --- | --- | --- | --- |
| *Fn*-Negative | 53 (61) | Ref |  |
| *Fn*-Low | 18 (21) | 0.29 (0.07, 1.10) | **0.07** |
| *Fn*-High | 16 (18) | 3.62 (0.82, 15.95) | **0.09** |

*^a^Fn* abundance was defined as the presence of detectable *Fn* DNA abundance (*Fn* positive) in fecal biospecimens. Patients were classified into *Fn* high and *Fn* low groups based on the median *Fn* Ct values specific to each study site (HD: low < 0.034, high > 0.034; HCI: low < 0.00067, high > 0.00067).

^b^Adjusted for age based on median age at diagnosis (<65 years old, ≥65 years old), stage at diagnosis (I-II, III), tumor site (colon, rectum), recruitment center (Heidelberg University Hospital, Germany or Huntsman Cancer Institute, Salt Lake City)
