# Supplementary material for: Fusobacterium nucleatum Abundance is Associated with Cachexia in Colorectal Cancer Patients: The ColoCare Study
Source: Cancer Med. 2024 Nov 25;13(22):e70431. doi: 10.1002/cam4.70431 (PMC11588854; doi:10.1002/cam4.70431)
Supplement: Supplementary file 2 — Supporting Information S1. [file CAM4-13-e70431-s001.docx]

**Supplemental Material**

***Fusobacterium nucleatum* abundance is associated with cachexia in colorectal cancer patients: The ColoCare Study**

Mmadili N. Ilozumba, PhD^1,2^_,_ Tengda Lin, MPH^1,2^, Sheetal Hardikar, MD, PhD^1,2^, Doratha A. Byrd, PhD^3^, June L. Round, PhD^1,4^, W. Zac Stephens, PhD^1,4^, Andreana N. Holowatyj, PhD^1,2,5^, Christy A. Warby, BA^1,2^, Victoria Damerell, PhD^6^, Christopher I. Li, PhD^7^, Jane C. Figueiredo, PhD^8^, Adetunji T. Toriola, MD, PhD^9^, David Shibata, MD^10^, Gary C. Fillmore, PhD^1^, Bartley Pickron, MD^1^, Erin M. Siegel, PhD^3^, Christoph Kahlert, MD^6^, Vaia Florou, MD, MS^1^, Biljana Gigic, PhD*******^6^, Jennifer Ose, PhD*******^1,2,11^, Cornelia M. Ulrich, PhD*******^1,2^

********These authors contributed equally and are co-last authors*

^1^Huntsman Cancer Institute, Salt Lake City, UT, USA,

^2^Department of Population Sciences, University of Utah, USA,

^3^H. Lee Moffitt Cancer Center and Research Institute, Tampa, FL, USA,

^4^Department of Pathology, Division of Microbiology and Immunology, University of Utah School of Medicine, Salt Lake City, UT, USA,

^5^Vanderbilt University Medical Center, Nashville, TN, USA,

^6^Department of General, Visceral, and Transplantation Surgery, Heidelberg University Hospital, Heidelberg, Germany,

^7^Fred Hutchinson Cancer Center, Seattle, WA, USA,

^8^Department of Medicine, Cedars-Sinai Medical Center, Samuel Oschin Comprehensive Cancer Institute, Los Angeles, CA, USA,

^9^Washington University School of Medicine in St. Louis, St. Louis, MO, USA,

^10^Department of Surgery, University of Tennessee Health Science Center, Memphis, TN, USA,

^11^Department of Information and Communication, Faculty for Media, Information and Design, University of Applied Sciences and Arts, Hannover, Germany

**Supplemental Methods**

**Study Population**

Study participants are from the prospective ColoCare Study (ClinicalTrials.gov NCT02328677), an international cohort of newly diagnosed stage I-IV CRC patients (ICD-10 C18-C20).^1^ The ColoCare Study design has been described elsewhere.^1-3^ The present study comprises data collected from n=87 patients (39 cachectic and 48 non-cachectic) diagnosed with stage I-III CRC enrolled between October 2010 and March 2018 at two study sites at the Heidelberg University Hospital ([HD], Germany, n=58) and the Huntsman Cancer Institute ([HCI], Utah, USA, n= 29) with pre-surgery available stool samples who met the following inclusion criteria: (i) available pre-operative fecal biospecimens collected at the patients’ homes and immediately stored in RNA*later* (Sigma-Aldrich, Germany [HD] and Thermo-Fisher Scientific Inc., MA, USA [HCI]) at -80°C), (ii) If patients received neo-adjuvant treatment, stool samples were collected at least 2 weeks after completion of neo-adjuvant treatment and (iii) with no antibiotic use reported within 4 weeks of biospecimen collection. The study was approved by the institutional review boards of the respective institutions, and all patients provided written informed consent

**Experimental Methods**

DNA/RNA was extracted from a 200μl RNAlater/fecal sample using the AllPrep PowerViral DNA/RNA Kit (Qiagen Inc., USA) according to the manufacturer’s instructions, including 2 minutes of bead-beating at 4ºC with a Mini-Beadbeater-16 (BioSpec Products, Bartlesville, OK). Quantitative real-time PCR (qRT-PCR) was performed on 0.2μl of template DNA. Reactions were performed in 20μl reactions containing primer,^4^ and 1× final concentration PowerUp Sybr Green MasterMix (Thermo Fisher Scientific Inc., MA, USA). All reactions were performed in duplicate. A positive control consisting of 60 pooled CRC patient samples were also included in each qRT-PCR run. DNA amplification and detection was performed with the CFX96 Real-Time System C1000 Thermal Cycler (Bio-Rad, CA, USA) using the following conditions: for *Fn*: 2 minutes at 50°C, 2 minutes at 95°C, and 45 cycles of 15 seconds at 95°C, 15 seconds at 57 °C and 20 seconds at 72°C. For 16S rRNA: 2 minutes at 50°C, 2 minutes at 95°C and 32 cycles of 15 seconds at 95°C, 20 seconds at 55°C and 15 seconds at 72°C. Primer sets and concentrations used have been previously described.^4,5^ Cycle thresholding (Ct) was calculated with a detection level of Ct=50 (Bio-Rad, CA, USA).

**Statistical Methods**

Comparisons between cachectic vs. non-cachectic patients were first examined using Chi-square and Fisher’s exact tests for categorical variables. The independent variable i.e., the *Fn* abundance was modeled as (*Fn*-low/*Fn*-negative [reference], *Fn*-high /*Fn*-positive) while the dependent variable i.e. onset of cachexia was modeled as cachectic vs non-cachectic CRC patients. Physical activity was categorized as (≥8.75 MET hrs/week, <8.75 MET hrs/week), with 8.75 MET hrs/week reflecting the guideline of at least 150 minutes (2.5 hours) of moderate to vigorous activity recommended for cancer survivors.^2,6,7^ CRP was categorized as (>=10mg/L, <10mg/L) based on clinical cutoff of >=10mg/L which usually indicates inflammation.^8^ Dietary fiber intake was categorized as (low fiber, high fiber) based on USDA recommendation for dietary fiber intake for adults under 50 (25 grams of fiber per day for women and 38 grams for men) and adults over 50 (21 grams for women and 30 grams for men).^9^ The stepwise selection procedure was used to select the covariates that were adjusted in the model. The full list of variables for the stepwise selection include median age at diagnosis, gender, recruitment center, stage at diagnosis, tumor site, BMI, CRP, physical activity, dietary fiber intake and neoadjuvant treatment. The p-value threshold set for the variables were slentry = 0.15 and slstay =0.15. The stepwise procedure retained median age at diagnosis, recruitment center and tumor site. We additionally adjusted for stage because stage of CRC at diagnosis significantly impacts clinical outcomes and is critical from a clinical perspective. In sensitivity analysis, antibiotic use in the past year was added to the stepwise selection procedure and was retained in the model.

**References**

1. Ulrich CM GB, Böhm J, Ose J, Viskochil R, Schneider M, Colditz GA, Figueiredo JC, Grady WM, Li CI, Shibata D, Siegel EM, Toriola AT, Ulrich A. . The ColoCare Study: A Paradigm of Transdisciplinary Science in Colorectal Cancer Outcomes. *Cancer Epidemiol Biomarkers Prev* 2019;28(3):591-601. doi:10.1158/1055-9965.EPI-18-0773.

2. Himbert C SW, Gigic B, Hardikar S, Holowatyj AN, Lin T, Ose J, Swanson E, Ashworth A, Warby CA, Peoples AR, Nix D, Jedrzkiewicz J, Bronner M, Pickron B, Scaife C, Cohan JN, Schrotz-King P, Habermann N, Boehm J, Hullar M, Figueiredo JC, Toriola AT, Siegel EM, Li CI, Ulrich AB, Shibata D, Boucher K, Huang LC, Schneider M, Round JL, Ulrich CM. . Differences in the gut microbiome by physical activity and BMI among colorectal cancer patients. *Am J Cancer Res*. 2022;12(10):4789-4801.

3. Eisele Y, Mallea PM, Gigic B, et al. Fusobacterium nucleatum and Clinicopathologic Features of Colorectal Cancer: Results From the ColoCare Study. *Clin Colorectal Cancer*. Sep 2021;20(3):e165-e172. doi:10.1016/j.clcc.2021.02.007

4. Tunsjo HS, Gundersen G, Rangnes F, Noone JC, Endres A, Bemanian V. Detection of Fusobacterium nucleatum in stool and colonic tissues from Norwegian colorectal cancer patients. *Eur J Clin Microbiol Infect Dis*. Jul 2019;38(7):1367-1376. doi:10.1007/s10096-019-03562-7

5. Bacchetti De Gregoris T, Aldred N, Clare AS, Burgess JG. Improvement of phylum- and class-specific primers for real-time PCR quantification of bacterial taxa. *J Microbiol Methods*. Sep 2011;86(3):351-6. doi:10.1016/j.mimet.2011.06.010

6. Piercy KL, Troiano RP, Ballard RM, et al. The Physical Activity Guidelines for Americans. *JAMA*. Nov 20 2018;320(19):2020-2028. doi:10.1001/jama.2018.14854

7. Schmitz KH, Courneya KS, Matthews C, et al. American College of Sports Medicine Roundtable on Exercise Guidelines for Cancer Survivors. *Medicine & Science in Sports & Exercise*. 2010;42(7):1409-1426. doi:10.1249/MSS.0b013e3181e0c112

8. Mac Giollabhui N, Ellman LM, Coe CL, Byrne ML, Abramson LY, Alloy LB. To exclude or not to exclude: Considerations and recommendations for C-reactive protein values higher than 10 mg/L. *Brain Behav Immun*. Jul 2020;87:898-900. doi:10.1016/j.bbi.2020.01.023

9. Soliman GA. Dietary Fiber, Atherosclerosis, and Cardiovascular Disease. *Nutrients*. 2019;11(5)doi:10.3390/nu11051155
